# Comprehensive quality evaluation for oysters of geographical indication from Rushan in China: Characteristic profile of nutrition and flavor components

**DOI:** 10.1016/j.fochx.2025.102320

**Published:** 2025-02-25

**Authors:** Shanqin Huo, Jixing Peng, Xinnan Zhao, Yichen Lin, Haiyan Wu, Guanchao Zheng, Qianqian Geng, Mengmeng Guo, Zhijun Tan

**Affiliations:** a Key Laboratory of Testing and Evaluation for Aquatic Product Safety and Quality, Ministry of Agriculture and Rural Affairs, Yellow Sea Fisheries Research Institute, Chinese Academy of Fishery Sciences, Qingdao 266071, China; b College of Food Science and Technology, Shanghai Ocean University, Shanghai 201306, China; c State Key Laboratory of Mariculture Biobreeding and Sustainable Goods, Yellow Sea Fisheries Research Institute, Chinese Academy of Fishery Sciences, Qingdao 266071, China

**Keywords:** *Crassostrea gigas*, Nutrition quality, Flavor components, Comprehensive evaluation

## Abstract

To reveal the differences in nutritional quality among diploid, triploid, and fattening oysters, nutrient and taste-contributing compounds were analyzed. The results demonstrate that diploid oysters exhibited significantly higher meat yield (22.88 %), protein (48.27 ± 2.50 g/100 g), total fatty acids (3032.52 ± 518.16 mg/100 g), vitamin B, mineral and flavor compounds than triploid and fattening oyster (*P* < 0.05). In contrast, triploid oysters exhibited higher lipid (10.02 ± 0.60 mg/100 g) and fat-soluble vitamin, and fattening oysters have higher EAA content (12.31 ± 0.69 mg/100 g) (*P* < 0.05). The edible quality of the three oyster types was evaluated using PCA and their nutrition value was assessed based on the NRF index score. The consistent rank results of the above two evaluation methods were diploid > triploid > fattening. The research results will provide data for oyster grading evaluation standards, thereby promoting the nutritional and health-oriented transformation of the oyster industry.

## Highlights

• Panoramic analysis of nutrition and taste quality of 30 geographical oysters from Rushan.
• The test quality of oysters could be distinguished between different types of samples.
• The comprehensive evaluation method as PCA score was completed through 18 key indices.
• The analysis of NRF index is consistent with the PCA score results.
• The quality of Rushan oysters was ranked as diploid > triploid > fattening.

## 1 Introduction

Seafood contains rich essential nutrients and functional components for enhancing human health (Golden et al., 2021; Tacon & Metian, 2013). It is particularly noteworthy that the nutrient content in bivalve shellfish is significantly higher than that in other livestock and poultry, such as amino acids, unsaturated fatty acids (EPA, DHA), vitamin A and mineral elements (Ca, Fe, Zn, Se) (Golden et al., 2021; Venugopal & Gopakumar, 2017; Wright, Fan, & Baker, 2018). Oysters also contain a variety of bioactive compounds, such as active peptides, polysaccharides and Zn, which have antioxidant, anti-inflammatory, immunomodulatory, anticoagulant, anti-skin aging, and intestinal flora-improvement functions (Zhang, 2022). Cheng et al. (2021) isolated, purified and identified three novel anticoagulants from the pepsin hydrolysate of *C. gigas* through a combination of bioinformatics and enzymatic approaches, which could effectively prolong the coagulation time. Li et al. (2015) isolated and purified a polysaccharide, ORPp, from *Ostrea rivularis*. ORPp significantly inhibited the formation of serum MDA and increased the level of antioxidant enzyme activity and total antioxidant capacity in rats. Moreover, it significantly improved the quality of male rat reproductive organs, promoted sperm motility, and increased the number of epididymal spermatozoa. Oysters are widely distributed along most global coastlines and have a long history of cultivation (Botta, Asche, Borsum, & Camp, 2020). Global oyster production was dominated by China, followed by France, the US, South Korea, Japan and Canada (Botta et al., 2020). Among them, *C. gigas* is native to northeast Asia and is one of the most significant commercially utilized varieties and is abundant on the coasts of north China (Howarth, Scanes, Byrne, & Ross, 2024; Jiang et al., 2023). In 2023, oyster production in China was reported over 6.67 million tons, of which 1.48 million tons were produced in Shandong (China Fisheries Statistical Yearbook, 2024). Rushan is famous as the hometown of oysters and Rushan oyster was granted the China National Geographical Indication Trademark (Zhao et al., 2022).

Currently, the commercially available Rushan oysters mainly include diploid, triploid, and fattening. The diploid oysters naturally grow and reproduce with two complete chromosome sets (Qin et al., 2018). Triploid oysters are individuals with three complete chromosome sets, resulting from the hybridization of diploids and tetraploids. Research on triploid oysters has been carried out since the early 1980s (Stanley, Allen, & Hidu, 1981). Triploid oysters have been widely adopted in shellfish culture to avoid the decline in diploid oyster growth and quality associated with sexual maturation (Jiang et al., 2023; Stanley et al., 1981). With the continuous development of the oyster industry, transferred fattening of oysters has become a common farming method. Oyster fattening is a method of improving the growth rate and quality of oysters which involves relocating young oysters to another location for fattening cultivation (Yin et al., 2024). The nutritional value of fattening oysters may differ from locally farmed oysters due to variations in growth conditions and fattening methods. Several studies have examined oysters in terms of seasonal, variety, and monthly (Isono et al., 2020; Qin et al., 2018). Previous research reported the differences in non-volatile and volatile compounds in diploid and triploid oysters (Bi, Xue, Wen, Li, & Liu, 2023). In addition, our research group has analyzed Rushan oysters and examined the impact of different regions, culture methods and seasons on oysters (Zhao et al., 2022). However, there is limited research on the comparative nutritional components of diploid, triploid and fattening oysters with the same origins. Therefore, a comprehensive study of diploid, triploid and fattening oysters from Rushan was carried out to distinguish their differences in proximate composition, amino acids, fatty acids, vitamins, minerals, and flavor substances. Oyster quality evaluation model and comprehensive scoring criteria were developed and assessed using principal component analysis (PCA). Nutrient-rich food (NRF) is a new index used to evaluate the nutritional quality of foods, which is used to grade foods based on their nutrient content (Sluik, Streppel, Van Lee, Geelen, & Feskens, 2015). In this study, the NRF index was employed to validate the results of PCA. The research may provide valuable insights into oyster industry cultivation and contribute to the promotion of oysters in the market.

## 2 Materials and methods

### 2.1 Reagents and instruments

N-hexane, chloroform, acetonitrile, and ethanol (chromatographically pure) were purchased from Merck & Co Inc. (Rahway, NJ, Germany); methanol, formic acid, acetic acid, ammonium formate, and ammonium acetate (mass spectrometry pure) were purchased from Fisher Scientific (Waltham, MA, USA); hydrochloric acid, boron trifluoride, sodium hydroxide, sodium chloride, sulfuric acid, nitric acid, and magnesium chloride (chemically pure) were purchased from Sinopharm Chemical Reagent Co. Ltd. (Shanghai, China).

20 amino acids standards (purity ≥99 %) and their internal standards mixture-^13^C,^15^N (767964-1EA) were purchased from Dr. Ehrenstorfer (Augsburg, Germany) and Sigma-Aldrich (Darmstadt, Germany), respectively. The standards of taurine, succinic acid, malic acid, citric acid, 6 vitamin B (B_1_-B_3_, B_5_-B_7_), and vitamin E (purity ≥99 %) were obtained from Dr. Ehrenstorfer (Augsburg, Germany). The standards of nucleotide (AMP, IMP, GMP), three vitamins (A, D_2_, D_3_, purity ≥99 %), and fatty acid standards (37 component mix, total 10 mg/mL) were purchased from ANPLE (Shanghai, China). The multi-element mixed standard was purchased from the China National Center for Standard Materials (Beijing, China).

### 2.2 Sample collection

**Fig. 1:**
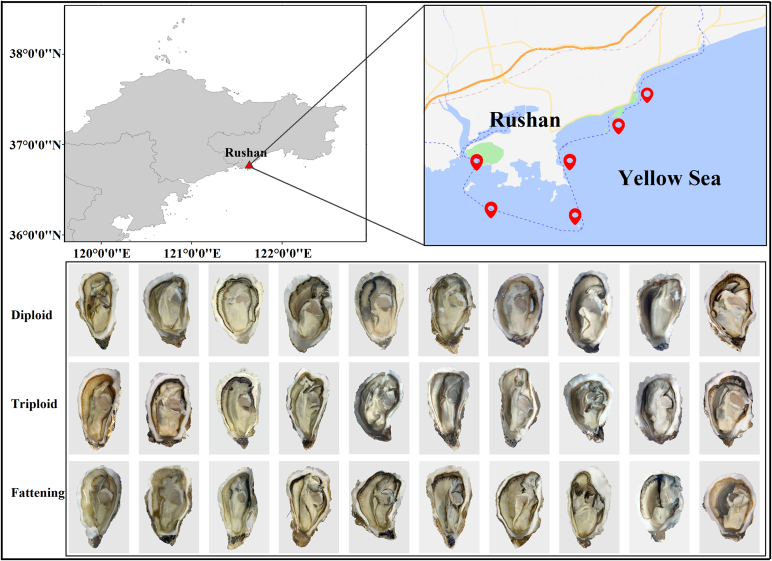
Sampling location and information of oysters from three types in Rushan.

In this study, 3 different types of oysters including diploid, triploid and fattening were collected in Rushan Bay, south of the Yellow Sea in April 2023 (Fig. 1). Diploid and triploid oysters are locally cultured in Rushan, while fattening oysters are transported from Rongcheng to Rushan for cultivation. Each species consisted of ten groups (*n* = 10), each containing about 3 kg of the same size individuals. All the live samples were kept chilled and transported to the laboratory, and biometric characteristics such as individual shell length, shell width, shell height, individual total weight, and soft tissue weight were measured (Table S1). The soft tissue was divided into two portions. One portion consists of homogenized fresh samples used to determine moisture content and flavor substances, while the other part is freeze-dried and crushed for the determination of nutrient indices such as lipid, protein, fatty acids, amino acids, mineral elements, and vitamins. The samples were stored at −80 °C in a freezer for further analysis.

### 2.3 Methods of sample analysis

**Proximate composition, total amino acids, fatty acids and minerals**: they were determined according to the National Food Safety Standards of China (NSPRC), moisture was measured using the direct drying method (NSPRC, GB 5009.3–2016); lipid was determined using the Soxhlet extraction method (NSPRC, GB 5009.6–2016); protein was determined by the Kjeldahl method (NSPRC, GB 5009.5–2016); Total amino acids were performed using an amino acids analyzer method (NSPRC, GB 5009.124–2016); fatty acids were performed using gas chromatography normalization (NSPRC, GB 5009.168–2016); the contents of nine minerals were quantified via the inductively coupled plasma mass spectrometry (ICP-MS) method (NSPRC, GB 5009.268–2016); selenium (Se) content was determined using atomic fluorescence spectrometry (NSPRC, GB 5009.93–2017). The analysis of vitamins and flavor compounds was based on the methods reported by our research group (Song et al., 2023). The detailed information as follow. **Vitamins**: the extraction of water-soluble vitamins used 10 mL 0.1 % formic acid solution, and the extract was finally dissolved in 10 mM ammonium formate solution before injecting into the LC-MS on the Kinetex XB C18 column (2.1 × 100 mm, 2.6 μm). Fat-soluble vitamin analysis was performed on the same column, and the two mobile phase solvents were methanol containing 0.1 % formic acid. **Taste composition**: 0.20 g sample was mixed with 20 mL of 0.1 % formic acid, vortexed for 2 min, then shaken in a 75 °C water bath for 15 min and sonicated at 40 kHz for 10 min. The mixture was centrifuged at 4000 r/min for 10 min. Following centrifugation, 50 μL of the clear supernatant was mixed with 50 μL internal standard, diluted to 1 mL with 0.1 % formic acid, filtered through a 0.22 μm PES syringe filter, and 2 μL mixture was injected into the Sciex 5500 Qtrap mass spectrometer system. Chromatographic separation was performed on an intrada amino acid column (3 × 100 mm, 3.0 μm) for analyzing free amino acids. Waters ACQUITY UPLC HSS T3 column (2.1 × 100 mm, 1.8 μm) was employed to determine nucleotides and organic acids. All analyses were performed in triplicate (*n* = 10).

### 2.4 Data analysis

According to the previously reported method, the meat yield was performed as described (Loaiza, Wong, & Thiyagarajan, 2023); atherogenicity index (AI) and thrombosis index (TI) were performed as described by Wang et al. (2023); ratio coefficient (RC), essential amino acid index (EAAI), and score of ratio coefficient of amino acid (SRC) were calculated according to the FAO/WHO recommended amino acid scoring pattern (FAO, 2024; Wang et al., 2023); mineral and vitamin composition was assessed using the Index of Nutrition Quality (INQ) (Song et al., 2023); Equivalent Umami Concentration (EUC) calculation method was based on the method by Song et al. (2023). The above indices are calculated using eqs. (1)–(9).

(1) $$\text{Meat yield}=\frac{\text{Soft tissue weight}}{\text{Total weight}}\times 100\%$$

(2) $$\mathrm{AI}=\left(C12:0+4\times C14:0+C16:0\right)/\left(\text{ΣMUFAs}+\text{ΣPUFAs}\right)$$

(3) $$\mathrm{TI}=\frac{\left(C14:0+C16:0+C18:0\right)}{\left\{0.5\times \text{ΣMUFAs}+0.5\times \mathrm{\Sigma n}-6\text{PUFAs}+3\times \mathrm{\Sigma n}-3\text{PUFAs}+\left(n-3/n-6\right)\right\}}$$

(4) $$\text{Amino acid scoring pattern}\;\left(\mathrm{AAS}\right)=\mathrm{mg}\;\text{of}\;\mathrm{EAA}\;\text{in}\;1\;g\;\text{of protein of test samples}/\mathrm{mg}\;\text{of}\;\mathrm{EAA}\;\text{in}\;1\;g\;\text{of protein of}\;\left(\mathrm{FAO}/\mathrm{WHO}\right)\;\text{reference pattern}\times 100$$

(5) $${\mathrm{RC}}_{k}={\mathrm{AAS}}_{k}/{\mathrm{AAS}}_{\text{mean}}$$

(6) SRC=100–RSD

(7) $$\text{EAAI}=\sqrt[{n}]{\left({\mathrm{Leu}}^{a}/{\mathrm{Leu}}^{b}\right)\times \left({\mathrm{Val}}^{a}/{\mathrm{Val}}^{b}\right)\times \ldots \times \left({\mathrm{Lys}}^{a}/{\mathrm{Lys}}^{b}\right)\times ({\mathrm{His}}^{a}/{\mathrm{His}}^{b}})\times 100$$

(8) INQ=consumed amount ofanutrientper1000kcal/recommended dietary allowance or adequate intake of that nutrientper1000kcal

(9) $$\mathrm{EUC}={\mathrm{\Sigma a}}_{i}b_{i}+1218\times \left({\mathrm{\Sigma a}}_{i}b_{i}\right)\times \left({\mathrm{\Sigma a}}_{j}b_{j}\right)$$

where MUFA means monounsaturated fatty acid and PUFA means polyunsaturated fatty acid; AAS_k_ is the amino acid ratio of the K essential amino acid in the sample; AAS_mean_ refers to the average value of all AAS_k_ in one sample; RSD is the variation number of RC; Leu^a^, Val^a^, …, His^a^ is the EAA concentrations of samples; Leu^b^, Val^b^, …, His^b^ is the EAA concentrations of standard pattern; n is the number of essential amino acids; a_i_ is the content of umami amino acids (Glu and Asp), g/100 g; b_i_ is the relative freshness coefficient relative to MSG (Glu is 1, Asp is 0.077). a_j_ is the content of tasty nucleotide, g/100 g; b_j_ is the relative freshness coefficient relative to IMP (IMP is 1, GMP is 2.3, AMP is 0.18). 1218 is the synergistic coefficient.

### 2.5 Statistic analysis

The mean ± standard deviation is used to express all experimental results. Data statistics were performed using Excel 2016 software, and one-way analysis of variance (ANOVA) was performed using SPSS 27.0 software. Multiple comparison was carried out using Duncan's test, with *P* < 0.05 regarded as a significant difference. Origin 2022 was used for data plotting and hierarchical cluster diagram. Pearson correlation analysis and PCA were performed using SPSS 27.0. Multivariate data analysis software SIMCA 14.1 (Umetrics Malmo, Scania, Sweden) was used to perform orthogonal partial least squares-discriminant analysis (OPLS-DA).

## 3 Results and discussion

### 3.1 Meat yield and proximate composition

**Fig. 2:**
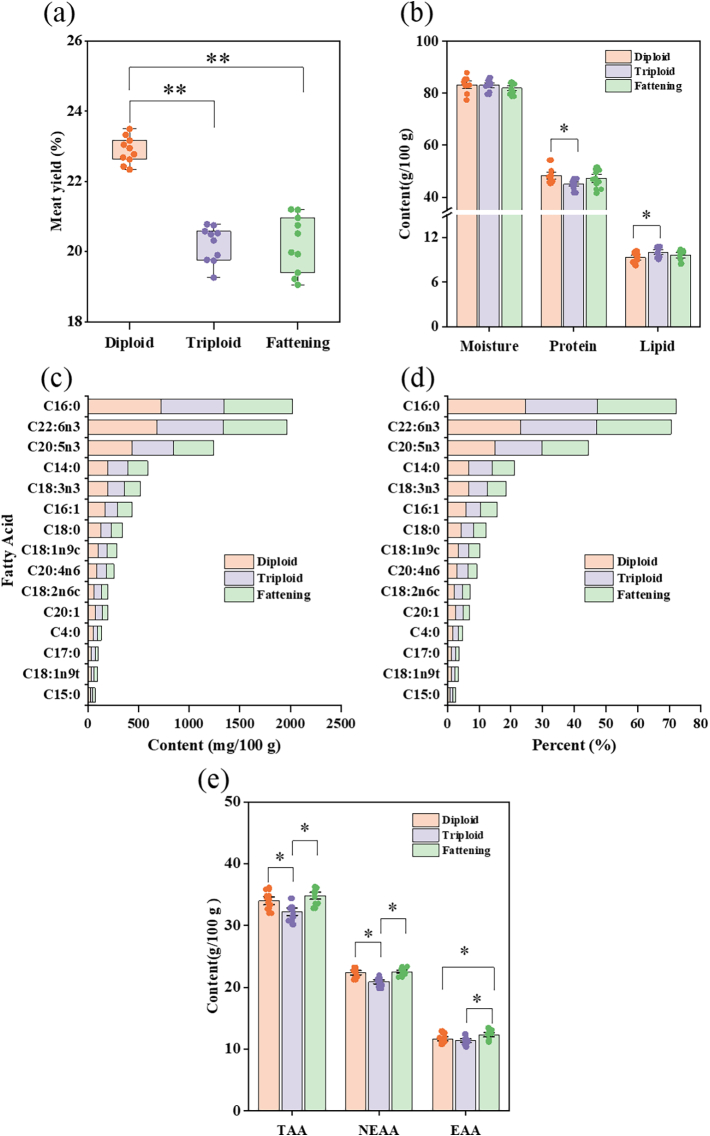
Difference analysis of nutritional quality in oyster sample. (a) Meat yield; (b) Proximate composition; (c) Fatty acid content; (d) Fatty acid compositions; (e) Amino acid. (dry weight). * and ** represent *P* < 0.05 and *P* < 0.01, respectively.

Fig. 2a and Table S2 display the meat yield of diploid, triploid, and fattening oyster. In this study, the meat yield of the oysters was approximately 20 %. The meat yield of *C. gigas* is greater than that of *C. ariakensis* and *C. angulata* (Loaiza et al., 2023). The entire soft tissue constitutes the edible portion of oysters after removing the shell. For oysters of the same size, the meat yield of diploid oysters (22.88 %) significantly exceeds that of triploids (20.21 %) and fattening oysters (20.22 %) (*P* < 0.01). However, the meat yield of triploid and fattening oysters is not significantly different (*P* > 0.05). Meat yield is one of the important indices for measuring the quality and production performance of aquatic products such as shrimp and shellfish, and can vary depending on growth environment, nutritional supplementation, genetic factors, and physiological conditions (Wu et al., 2020).

When assessing whether the nutritional value of seafood satisfies dietary requirements and commercial quality standards, it is essential to evaluate its proximate composition. In this study, the moisture, protein, and lipid content of three types of *C. gigas* are shown in Fig. 2b. The moisture content ranges from 81.99 g/100 g to 83.28 g/100 g; the protein content ranges from 45.02 g/100 g to 48.27 g/100 g; and the lipid content ranges from 9.42 g/100 g to 9.80 g/100 g, which is consistent with previous studies (Zhao et al., 2022). There were no significant differences in moisture content among the three types of *C. gigas* (*P* > 0.05), while the protein content in diploids (48.27 ± 2.50 g/100 g) was significantly higher than that of triploids (45.02 ± 1.65 g/100 g) (*P* < 0.05). In contrast to protein, the lipid content was lower in diploids. Similar results were also observed in previous research (Fu et al., 2024). To maintain their essential physiological processes and metabolic activity during reproductive periods, diploid oysters may store more protein and energy (Joaquim, Matias, Lopes, Arnold, & Gaspar, 2008).

The content of high-quality proteins and lipids directly determines the nutritional value of the food, which significantly impacts its market value (Venugopal & Gopakumar, 2017). All three types of *C. gigas* have higher protein and lipid contents than other marine products, like crucian carp (Sun et al., 2022) and mackerel crabs (Cao et al., 2022), although triploid oysters have a lower protein level. Protein and lipid indices were selected for correlation analysis (CA) and hierarchical cluster analysis (HCA).

### 3.2 Fatty acid content

The three types of oysters were found to contain 15 types of fatty acids: 6 types of saturated fatty acids (SFA), 4 types of monounsaturated fatty acids (MUFA), and 5 different types of polyunsaturated fatty acids (PUFA). The fatty acid content in different types is shown in Table S3 and Fig. 2c-d. In this study, the content ranges of SFA, MUFA, and PUFA in *C. gigas* were 1027.72–1161.78 mg/100 g, 301.65–385.58 mg/100 g, and 1322.00–1464.01 mg/100 g, respectively. Diploid oysters exhibit the highest levels of SFA (1161.78 ± 38.86 mg/100 g), MUFA (385.58 ± 9.03 mg/100 g), and PUFA (1464.01 ± 26.99 mg/100 g) in *C. gigas* (*P* < 0.05). The differences in fatty acid composition of different types of *C. gigas* may be related to linoleic acid metabolism, the biosynthesis of unsaturated fatty acids, and the biosynthesis of pantothenic acid and CoA (Bao et al., 2023).

The composition and amount of unsaturated fatty acids are key indications for assessing the nutritional value of seafood, and they have a variety of active roles (Wang et al., 2023). Previous studies suggest that marine shellfish can serve as a valuable supply of omega-3 polyunsaturated fatty acids, including docosahexaenoic acid (DHA) and eicosapentaenoic acid (EPA), which are crucial for maintaining human health (Venugopal & Gopakumar, 2017). In this study, the EPA + DHA content of *C. gigas* ranged from 1025.61 to 1118.67 mg/100 g, surpassing the recommended daily intake of 500 mg EPA + DHA per individual (Casula, Soranna, Catapano, et al., 2013). Diploid oysters have the highest EPA + DHA content, followed by triploids, and lowest in fattening oysters. This could be because PUFA levels are significantly impacted by variations in living conditions and food sources (Sushchik, Gladyshev, & Kalachova, 2007).

The n-3/n-6 ratio is an important index to assess the nutritional value of food, which can reflect whether the fatty acid content in diet is suitable (Wang et al., 2023). A dietary n-3/n-6 ratio range of 5:1 to 10:1 is advised by the FAO; the higher the ratio, the higher the nutritional value of the food (FAO, 2024). In this study, *C. gigas* were found to have an n-3/n-6 ratio ranging from 7.23 to 8.69. This implies that oysters are an excellent source of n-3 unsaturated fatty acids. The n-3/n-6 ratio in diploid and fattening oysters was significantly greater than that in triploid oysters (*P* < 0.05). This difference may be attributed to the higher levels of EPA and alpha-linolenic acid in diploid oysters. AI and TI are used to assess the effects of dietary fatty acid components on human health. The AI and TI indices are negatively correlated with human health, with recommended values of less than 1.0 and 0.5, respectively (Fernandes et al., 2014). The results of this study showed that the fatty acid composition of *C. gigas* is appropriate, with the AI of the various types ranging from 0.83 to 0.88 and the TI from 0.23 to 0.26. Overall, there are differences in the fatty acid content of the three oyster types. Thus, EPA + DHA, SFA, MUFA, PUFA, and n-3/n-6 indices were selected for CA and HCA.

### 3.3 Amino acid content

Amino acids are the basic components of proteins and peptides, which play a crucial role in metabolic processes. The nutritional value of amino acids mainly depends on the types, quantities, and composition of essential amino acids (EAA) (Wright et al., 2018). 18 amino acids were detected in different types of *C. gigas* (Table S4), with the total amino acid (TAA) content ranging from 32.23 to 34.82 g/100 g. The composition of EAA is an important index for evaluating the nutritional value of aquatic protein. In this study, the total essential amino acid content in *C. gigas* was 11.35–12.31 g/100 g, accounting for approximately 35 % of the total amino acids (Fig. 2e). The trends in TAA and non-essential amino acids (NEAA) content were consistent with the protein content in oysters, whereas the EAA of the fattening oysters was significantly higher than those of the diploid and triploid oysters (*P* < 0.05). This may be related to the synthesis and catabolism of proteins (Bao et al., 2023). Diploid oysters exhibit a high rate of protein synthesis and a relatively low rate of decomposition, resulting in a high protein content. According to the WHO, the recommended ideal protein pattern specifies that the standard value for EAA/TAA is approximately 0.4. In this study, the EAA/TAA of the three types of *C. gigas* is approximately 0.35, suggesting that *C. gigas* is a superior source of protein that can more effectively meet the dietary requirements of humans.

The closer the EAA composition of protein in food is to the human body, the higher its protein quality. Thus, RC, SRC, and EAAI are employed in this study to assess the quality of the protein (Lin et al., 2025). The analysis findings are displayed in Table 1. The closer the RC value in food is to 1, the more the food meets human requirements. Whereas RC value >1 shows that the diet is rich in that amino acid, RC value <1 suggests that the amino acid is a limiting amino acid (Wang et al., 2023). In this study, the first limiting amino acid for different types of *C. gigas* was valine, which is similar to previous research (Qi et al., 2019). EAAI is an important index for evaluating the nutritional value of proteins. The nutritional value of protein is positively correlated with EAAI value. The EAAI of different types of *C. gigas* ranges from 63.25 to 68.47, with fattening oysters exhibiting higher EAAI values than diploid and triploid oysters (*P* < 0.05). The SRC is used to evaluate the nutritional value of proteins in food. The closer the SRC value is to 100, the more balanced the essential amino acids profile in the food, and the higher its nutritional value. The SRC of *C. gigas* is 95.12–96.19, indicating that *C. gigas* protein has a high biological value, close to the recommended levels of essential amino acids for human diets. Therefore, the representative indices of EAA, TAA and EAAI were selected for subsequent analysis.

**Table 1:** Evaluation of essential amino acid composition of oyster samples (dry weight).

Indices	FAO/WHO (mg/g pro)	Diploid		Triploid		Fattening	
		AAS	RC	AAS	RC	AAS	RC
Ile*	40	1.38	1.29	1.43	1.28	1.47	1.27
Leu*	70	1.02	0.96	1.07	0.96	1.10	0.95
Lys*	55	0.88	0.82	0.92	0.82	1.01	0.87
Met* + Cys	35	1.16	1.08	1.22	1.09	1.23	1.06
Phe* + Tyr	60	1.35	1.26	1.41	1.26	1.44	1.23
Thr*	40	1.07	1.00	1.10	0.99	1.19	1.03
Val*	50	0.64	0.60	0.67	0.60	0.68	0.58
EAAI		63.25		66.04		68.47	
SRC		96.63		95.82		95.40	

Note: * represents essential amino acid.

### 3.4 Vitamin and mineral content

**Fig. 3:**
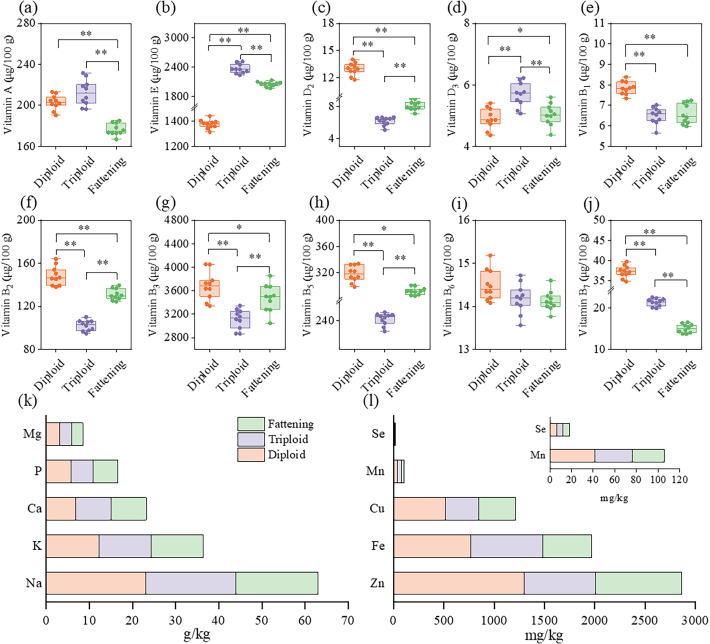
Difference analysis of vitamins and minerals in oyster sample. (a-j) Vitamin contents; (k-l) Mineral contents. (dry weight). * and ** represent *P* < 0.05 and *P* < 0.01, respectively.

Vitamins are essential for maintaining normal physiological metabolism and health, and their content is an important dimension reflecting the nutritional value of oysters (Golden et al., 2021). In this study, 4 kinds of fat-soluble vitamins (vitamin A, E, D_2_, D_3_) and 6 water-soluble vitamins (B_1_-B_3_, B_5_-B_7_) were measured and the contents were consistent with previous investigations (Venugopal & Gopakumar, 2017; Wright et al., 2018). The fat-soluble vitamins A and E have relatively high levels, ranging from 171.51 μg/100 g to 212.34 μg/100 g and from 1.30 mg/100 g to 2.36 mg/100 g, respectively. The content of water-soluble vitamins B_2_, B_3_, and B_5_ is also relatively high, with ranges of 102.19 μg/100 g to 148.32 μg/100 g, 3.11 mg/100 g to 3.68 mg/100 g, and 240.44 μg/100 g to 318.46 μg/100 g, respectively. Table S5 and Fig. 3a-j reveal that the diploid oysters had the highest content of the other five water-soluble vitamins (*P* < 0.05), with no significant difference observed for vitamin B_6_ (*P* > 0.05).

Minerals are a class of indispensable substances that participate in various physiological processes of the human body. Mineral elements, including macro elements and trace elements, cannot be synthesized within the human body and just be obtained through diet, which are essential for human growth and development, and play an important role in various metabolic activities and biochemical processes (Nędzarek & Czerniejewski, 2021). According to a report by Tacon and Metian, aquatic foods are richer in most essential elements than those in most terrestrial meats (Tacon & Metian, 2013). In this study, five macro elements (K, Ca, Na, Mg, P) and five trace elements (Fe, Cu, Mn, Zn, Se) were measured in *C. gigas* (Table S6 and Fig. 3k-l). The highest amounts of Na, Mg, Fe, Zn, Cu, and Mn were found in the diploid oysters (*P* < 0.05), whereas fattening oysters exhibited the highest levels of Ca (*P* < 0.05). The levels of Fe, Mn, Se and Cu are consistent with previous studies (Lin et al., 2019). Diploid and fattening oysters have a greater P content than triploid oysters (*P* < 0.05). The K and Se content showed no significant differences among the three types (*P* > 0.05). The differences in mineral content may be related to the living environment and food sources of the different types of *C. gigas* (Wang et al., 2020).

The INQ was employed to evaluate the nutritional value of vitamins and minerals. The results are shown in Table S7. The INQ values of vitamins A, D, B_3_, and B_7_ exceed 1, indicating that these four vitamins are the dominant nutrients in *C. gigas*. The vitamins with the highest INQ values are vitamin D and vitamin B_7_ (10.26–15.53 and 3.25–8.08, respectively), indicating that vitamin D and vitamin B_7_ are characteristic and advantageous essential nutrients in *C. gigas*, and can serve as high-quality food sources for specific populations to supplement vitamin. The INQ values of the mineral elements measured in *C. gigas* exceed 1, indicating that *C. gigas* are a good source of minerals, with the highest INQ values for Mn, Zn, and Se. Therefore, the indices of vitamin B_3_, B_5_, B_7_, A, D, E, Ca, Fe, Zn and Se were selected for CA and HCA.

### 3.5 Flavor substances and taste activity value (TAV)

The unique taste of *C. gigas* origins from its flavor amino acids, nucleotides, and organic acids (Song et al., 2023; Wang et al., 2023). Thus, non-volatile flavor substances were tested in oyster samples, which included 18 free amino acids (including taurine, total content 1270–1410 mg/100 g), 3 nucleotides (IMP, AMP, GMP, total content 11.26–19.88 mg/100 g), and 3 organic acids (succinic, malic and citric, total content 53.10–101.64 mg/100 g). The total amounts of these three types of flavor substances showed the same trend: diploid > triploid > fattening (Table 2).

**Table 2:** Flavoring substances contents of oyster samples (wet weight).

Flavor substances (mg/100 g)	Taste attribute	Taste threshold (mg/100 g)	Content (mg/100 g)			TAV		
			Diploid	Triploid	Fattening	Diploid	Triploid	Fattening
Glutamic Acid	Umami (+)	30	150.13 ± 4.10^a^	141.35 ± 6.34^b^	131.60 ± 9.56^c^	5.00	4.71	4.39
Aspartic Acid	Umami (+)	100	36.48 ± 3.19^a^	24.58 ± 2.49^b^	21.99 ± 3.97^b^	0.36	0.25	0.22
Glycine	Sweet (+)	130	118.81 ± 3.49^a^	113.93 ± 2.42^b^	108.53 ± 3.93^c^	0.91	0.88	0.83
Alanine	Sweet (+)	60	107.43 ± 4.78^a^	85.94 ± 3.34^b^	87.23 ± 2.80^b^	1.79	1.43	1.45
Serine	Sweet (+)	150	29.67 ± 1.69^b^	34.05 ± 2.95^a^	30.44 ± 2.70^b^	0.20	0.23	0.20
Threonine	Sweet (+)	260	34.94 ± 2.98^a^	27.36 ± 2.82^b^	29.19 ± 3.46^b^	0.13	0.11	0.11
Proline	Sweet/bitter (+)	300	75.38 ± 4.13^a^	68.99 ± 5.03^a^	69.75 ± 4.24^a^	0.25	0.23	0.23
Arginine	Sweet/bitter (+)	50	100.55 ± 6.20^a^	92.53 ± 6.44^b^	85.49 ± 3.20^c^	2.01	1.85	1.71
Methionine	Bitter/Sweet/ sulfurous (−)	30	17.60 ± 2.03^a^	15.97 ± 5.07^a^	14.63 ± 2.07^a^	0.59	0.53	0.49
Tryptophan	−	−	7.06 ± 0.83^a^	5.62 ± 0.45^b^	5.74 ± 0.50^b^	−	−	−
Tyrosine	−	−	15.99 ± 0.73^a^	14.31 ± 0.56^b^	14.21 ± 0.82^b^	−	−	−
Histidine	Bitter (−)	20	16.79 ± 2.30^ab^	17.28 ± 1.36^a^	15.23 ± 1.67^b^	0.84	0.86	0.76
Leucine	Bitter (−)	190	26.30 ± 1.18^b^	26.28 ± 2.54^b^	29.04 ± 3.51^a^	0.14	0.14	0.15
Isoleucine	Bitter (−)	90	9.61 ± 1.63^a^	8.69 ± 0.70^a^	7.21 ± 1.52^b^	0.11	0.10	0.08
Phenylalanine	Bitter (−)	90	15.34 ± 1.56^a^	13.67 ± 2.86^a^	15.00 ± 2.14^a^	0.17	0.15	0.17
Valine	Sweet/bitter (−)	40	14.85 ± 2.58^a^	15.08 ± 1.00^a^	12.08 ± 1.42^b^	0.37	0.38	0.30
Lysine	Sweet/bitter (−)	50	114.70 ± 5.27^c^	129.64 ± 2.88^b^	143.64 ± 5.98^a^	2.29	2.59	2.87
Taurine	−	–	518.44 ± 13.38^a^	461.89 ± 15.54^b^	449.07 ± 16.06^b^	−	−	−
IMP	Umami (+)	25	5.32 ± 0.35^a^	4.82 ± 0.29^b^	3.43 ± 0.36^c^	0.21	0.19	0.14
AMP	Umami/Sweet (+)	50	13.51 ± 0.44^a^	11.76 ± 0.88^b^	7.18 ± 0.55^c^	0.27	0.24	0.14
GMP	Umami (+)	12.5	1.05 ± 0.06^a^	1.01 ± 0.04^b^	0.65 ± 0.04^c^	0.08	0.08	0.05
Succinic	Sour/umami	10.6	46.73 ± 3.53^a^	27.73 ± 2.59^a^	27.18 ± 2.26^a^	4.41	2.91	2.24
Malic	Sour/bitter	49.6	30.86 ± 1.45^b^	20.82 ± 1.96^b^	15.29 ± 1.34^b^	0.56	0.42	0.31
Citric	Sour	45	23.73 ± 1.67^c^	15.18 ± 1.39^c^	14.19 ± 1.36^b^	0.60	0.34	0.32

Note: Values are presented as means ± SD.

Means with different letters are significant differences (*P* < 0.05) and means with the same letters are not significant differences (*P* > 0.05).

**Fig. 4:**
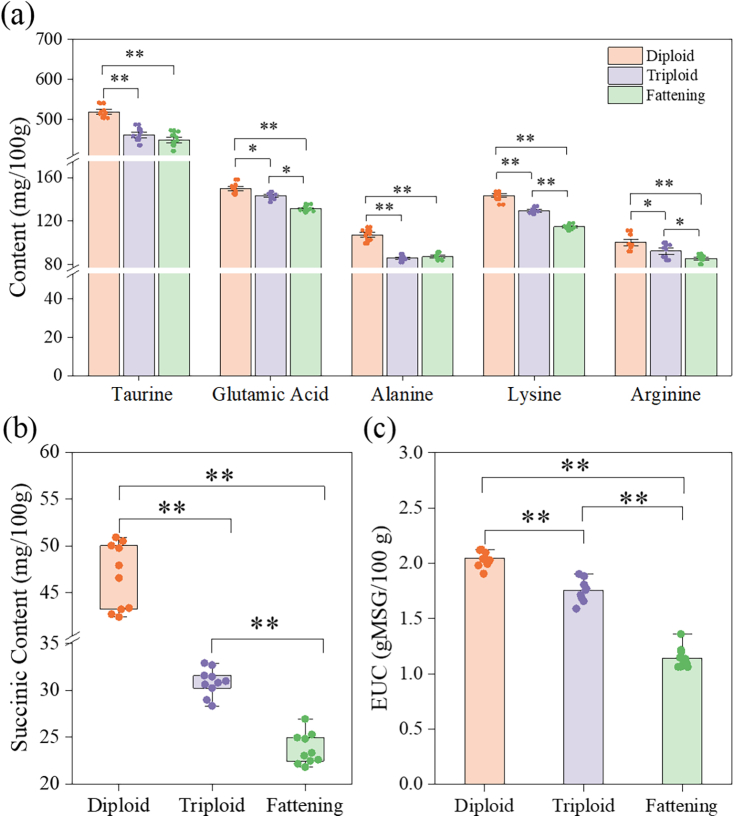
Difference analysis of flavor substances in oyster sample. (a) Taurine and the content of important taste substance (TAV > 1); (b) Succinic content; (c) EUC value. * and ** represent *P* < 0.05 and *P* < 0.01, respectively.

Taurine not only has specific physiological functions but also imparts a unique flavor to food (Isono et al., 2020). Previous studies have demonstrated that oysters are a rich source of taurine (Zhu, Li, Yu, & Kong, 2018). In this study, the taurine content accounted for approximately 35 % of the total free amino acids, and the taurine content in diploid oysters was significantly greater than that in triploid and fattening oysters (*P* < 0.05) (Fig. 4a). Furthermore, the three types of *C. gigas* are also rich in glutamic acid, lysine, glycine, proline, and arginine, which is consistent with previous researches (Song et al., 2023; Wang et al., 2023; Zhu et al., 2018). AMP has been shown to have an excellent flavor-enhancing impact on shellfish, significantly promoting umami and inhibiting bitterness to some extent (Kim, Son, Kim, Misaka, & Rhyu, 2015). According to this study, the flavor of oysters can be improved by the presence of umami amino acids, despite the relatively low concentration of these nucleotides in oysters (Lin et al., 2025). Succinic acid is the main flavoring organic acid in *C. gigas*, with the highest content in diploid oysters (46.73 ± 3.53 mg/100 g) (*P* < 0.05) (Fig. 4b).

However, the human perception of taste is influenced by both the content of taste-active substances and their taste thresholds (Kawai, Okiyama, & Ueda, 2002). When TAV is greater than 1, it indicates that the food has taste activity; the higher the TAV, the greater its contribution to taste (Bi et al., 2023). The flavor substances with a TAV > 1 include glutamic acid, alanine, arginine, lysine, and succinic acid. Glutamic acid and succinic acid are the primary umami enhancers in oysters, while glycine promotes sweetness, and lysine has certain inhibitory effects on taste. When the TAV of arginine exceeds 1, arginine produces a bitter taste, which may mask its sweetness at lower concentrations (Wang et al., 2023). Sodium succinate plays a crucial role in the taste of mollusks because phosphoenolpyruvate formed through glycolysis causes the accumulation of succinate in clams and scallops via the tricarboxylic acid cycle (Zhu et al., 2023). In comparison to scallops and abalone, the TAV value of alanine in oysters was higher (Song et al., 2023; Wang et al., 2023).

To comprehensively evaluate the flavor substance differences among different types of *C. gigas*, the EUC values were calculated. The EUC value for the three types of *C. gigas* ranged from 1.14 to 2.04 (Fig. 4c). The EUC values show that diploid > triploid > fattening, indicating that diploid oysters have a superior taste compared to the other two types.

### 3.6 Pearson correlation analysis and hierarchical cluster analysis (HCA)

**Fig. 5:**
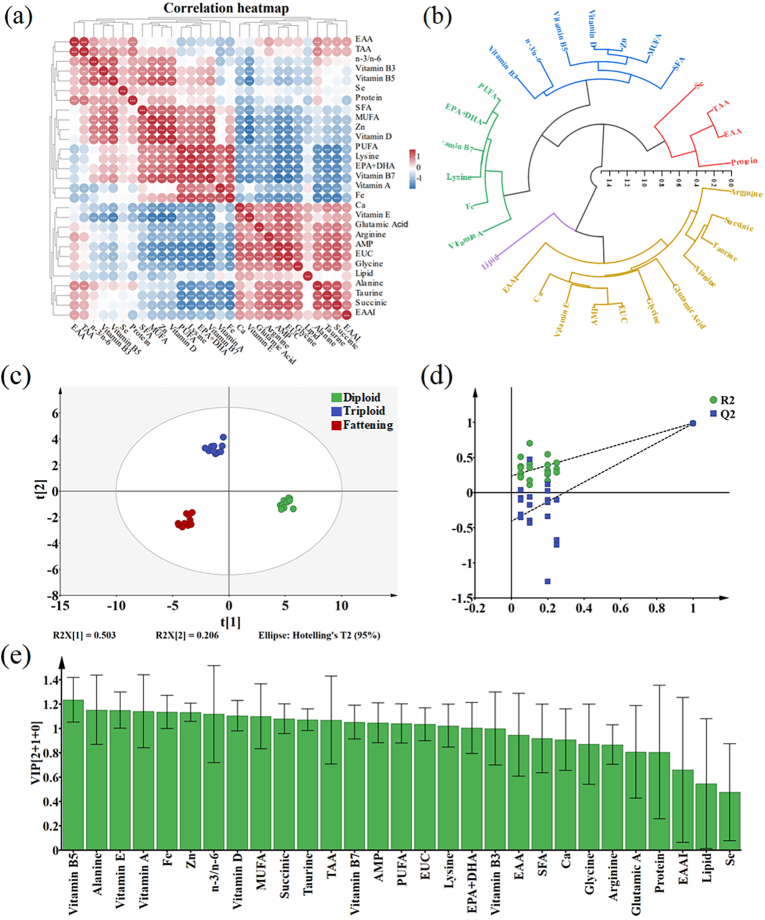
Multivariate statistical analysis of oyster samples. (a) Pearson correlation heatmap; (b) Hierarchical cluster diagram of 29 quality indices. (c) OPLS-DA score; (d) VIP value; (e) Permutation test plot. “*” indicates significant correlation (*P* < 0.05).

A total of 29 indices were analyzed, including the proximate composition (protein, lipid); nutritional substances (EAAI, EAA, TAA, EPA + DHA, SFA, MUFA, PUFA, n-3/n-6, Ca, Fe, Zn, Se, vitamin B_3_, B_5_, B_7_, A, D, and vitamin E) and flavor components (glutamic acid, alanine, arginine, glycine, lysine, taurine, AMP, succinic acid, and EUC) were selected for CA. The Pearson correlation coefficients ranging from −1 to 1 and two-tailed tests were calculated for *C. gigas* nutrition indices. Meanwhile, significant correlations were marked by an asterisk(*) (Xu et al., 2024). The correlation results are shown in Fig. 5a. To avoid information duplication, it is necessary to simplify the pre-evaluation of oysters due to the variety of quality indices and their interrelationships (Xu et al., 2024). Using hierarchical cluster analysis (HCA), a hierarchical graph represents the similarity among classes, where a smaller distance between data points indicates a higher degree of similarity (Xu et al., 2024). The classification boundaries become more discernible by starting with the most similar objects and methodically grouping them into a unified class (Xu et al., 2024). As shown in Fig. 5b, in accordance with the Euclidean distance of 0.7, 29 quality indices of oysters are classified into 5 categories (Xu et al., 2024). CA and HCA revealed varying degrees of correlation among nutrition indexes, some of which showed significant correlation. The correlation among indices is complex and the information overlaps, leading to redundancy. Therefore, these indexes cannot directly and accurately evaluate the comprehensive quality of different types of oysters, and further comprehensive analysis should be carried out by multivariate statistical methods.

### 3.7 Orthogonal partial least squares discriminant analysis (OPLS-DA)

OPLS-DA is widely regarded as an effective method for establishing sample classification and discrimination models (Song et al., 2023). An OPLS-DA model was developed to screen the nutritional indices that contribute significantly to classification and determine the differential characteristic indices of several oyster types. The three types of *C. gigas* may be clearly distinguished from one another without overlapping, as seen in Fig. 5c, suggesting that their discrimination is superior. A total of 18 nutrition indices were screened by the variable importance in the projection (VIP) with scores above 1, as shown in Fig. 5e. The 18 indices included total amino acid (TAA), 4 fatty acids indices (EPA + DHA, MUFA, PUFA, n-3/n-6), 5 vitamins (vitamin A, D, E, B_5_, and vitamin B_7_), 2 minerals (Zn, Fe) and 6 flavor substances (alanine, lysine, AMP, succinic, taurine, EUC). In this study, after 200 permutation tests, the values of parameters the model R2X, R2Y, and Q2 were 0.781, 0.983, and 0.971, respectively, indicating that the model is reliable and has high predictability (Bao et al., 2023; Song et al., 2023) (Fig. 5d).

### 3.8 Principal component analysis (PCA)

The PCA comprehensive evaluation model can simplify the data collection and facilitate reliable quality evaluation for oysters based on the optimized key physicochemical indices. PCA factor analysis was performed on 18 core quality indices, as shown in Table 3. Based on the criteria of cumulative variance percentage greater than 80 % or eigenvalue greater than 1 (Kong et al., 2024), which led to the selection of two principal components. The variance contribution of the first principal component (PC1) was 59.36 %, with representative indices including PUFA、EPA + DHA、vitamin B_7_、lysine、AMP and EUC. The second principal component (PC2) accounted for 28.00 % of the total variance with representative indices including n-3/n-6 and vitamin B_5_. The cumulative contribution rate reached 87.36 %, explaining most of the information on the 18 differential variables.

**Table 3:** 18 quality indices of principal component factor analysis of oyster samples.

Index	PC1	PC2
TAA	−0.055	0.773
MUFA	0.783	0.505
PUFA	0.911	−0.299
EPA + DHA	0.935	−0.130
n-3/n-6	0.279	0.841
Fe	0.808	−0.525
Zn	0.852	0.499
Vitamin A	0.501	−0.742
Vitamin D	0.843	0.459
Vitamin E	−0.802	−0.568
Vitamin B_3_	0.439	0.668
Vitamin B_5_	0.517	0.813
Vitamin B_7_	0.988	0.031
Alanine	−0.659	0.675
Lysine	0.916	−0.235
Taurine	−0.772	0.486
AMP	−0.978	0.018
Succinic	−0.834	0.434
EUC	−0.969	−0.004
Eigenvalue	11.278	5.320
VCR(%)	59.357	28.000
CVCR(%)	59.357	87.357

Note: VCR represents percentage of variance, CVCR represents cumulative percentage of variance.

Using the variance contribution rate of each principal component as the weight, the comprehensive evaluation score function of each oyster type was established according to the principal component factor score formula. The function is represented as follows: F = 0.679 × F1 + 0.321 × F2, where F represents the comprehensive score for each oyster type. The comprehensive quality ranks for three groups were calculated by the principal component comprehensive scoring model, which is shown in Table 4, and the order is diploid > triploid > fattening.

**Table 4:** Principal component factor scores and nutrient-rich food index scores of oyster samples.

**Groups**	Principal Component Factor Score				Nutrient-rich Food Index Score			
	F1	F2	F	Rank	NR	LIM	NRF	Rank
**Diploid**	2.93	2.47	2.785	1	445.83	30.49	415.34	1
**Triploid**	1.58	−2.91	0.143	2	346.08	28.28	317.81	2
**Fattening**	−4.52	0.44	−2.928	3	341.35	28.32	313.03	3

### 3.9 Nutrient-rich food (NRF) index

The NRF index is an evaluation of the comprehensive nutritional value of food, which can directly reflect the overall nutritional value of food (Streppel et al., 2014). The higher the index value, the higher the recommended nutrient content of oysters, and the better the nutritional quality; on the contrary, the lower the index value, the poorer the nutritional quality (Streppel et al., 2014). It is easy for consumers to understand, and it facilitates easier assessment of the nutritional value of food for consumers. The NRF index represents the difference between the nutrient quality index of the recommended nutrient (NR) and the nutrient quality index of the Limiting nutrients (LIM) (Sluik et al., 2015). Protein, n-6, n-3, EPA + DHA, minerals, and vitamins present in oysters were selected as recommended nutrients. The limiting nutrients include sodium and saturated fatty acids. The results are shown in Table 4, and the nutrient richness index values are ranked as diploid > triploid > fattening oysters.

Both PCA factor analysis and the NRF index are widely used in the comprehensive evaluation of food nutritional quality, and the two methods were mutually verified in this study. The results of both methods showed that diploid oysters had the best nutritional quality among the three groups.

In general, this study revealed the key quality characteristics of three types of oysters. In the oyster industry, the established evaluation model of oysters can be used to comprehensively evaluate the quality of oysters. Therefore, the results of this study can guide the quality improvement and high-value development of different types of oysters. However, further research is still needed for the future development of the oyster industry. At present, the metabolic mechanisms underlying oyster samples remain unclear, and the understanding of nutritional quality differences in oysters is still limited. Therefore, further studies on metabolic regulation of oyster samples are needed in the future. More advanced analysis techniques should be employed to further explore the mechanism of different factors affecting the nutritional quality of oysters.

## 4 Conclusion

The nutritional quality of diploid, triploid, and fattening oysters of the same sizes was assessed and compared in this study. The meat yield and protein contents of diploid oysters are higher than those of the other two types. EPA + DHA were the main fatty acid components, which is consistent with the health benefits of oysters as seafood is rich in essential fatty acids. The contents of SFA, MUFA and PUFA were higher in diploid oysters, but there were no differences in the fatty acid composition. The highest levels of vitamins B_3_, B_5_, and vitamin E are found, while the INQ values of vitamins D and B_7_ are relatively high. The *C. gigas* is a good source of minerals because all of the INQ values of mineral elements are higher than 1. Oysters are one of the most distinctive tasting shellfish, characterized by higher concentrations of umami-taste or sweet-taste free amino acids (such as glutamic acid and glycine), 5′-nucleotides (such as AMP), and organic acids (such as succinic). Using the comprehensive evaluation methodologies of OPLS-DA and PCA, the study ranked the oyster samples based on their nutritional and flavor qualities. At the same time, the NRF index was used to evaluate oysters, and the results were consistent with the PCA results. Three types of oysters were ranked as diploid > triploid > fattening. The results showed that diploid oysters have a more delectable taste and richer nutrition, followed by triploid and fattening oysters. The results of this study will provide scientific references for the culture, consumption, processing, nutritional quality evaluation of oysters, and mining of characteristic indices. At the same time, it also enhanced consumers' understanding of the comprehensive nutritional value evaluation of oysters, guided consumption practice from the perspective of nutrition and flavor, and provided basic data for further research on oysters.

## CRediT authorship contribution statement

**Shanqin Huo:** Writing – original draft, Methodology, Investigation, Formal analysis, Data curation, Conceptualization. **Jixing Peng:** Writing – review & editing, Project administration, Funding acquisition, Formal analysis. **Xinnan Zhao:** Methodology, Investigation, Data curation. **Yichen Lin:** Formal analysis. **Haiyan Wu:** Formal analysis. **Guanchao Zheng:** Formal analysis. **Qianqian Geng:** Formal analysis. **Mengmeng Guo:** Formal analysis. **Zhijun Tan:** Writing – review & editing, Supervision, Funding acquisition, Formal analysis.

## Declaration of competing interest

The authors declare that they have no known competing financial interests or personal relationships that could have appeared to influence the work reported in this paper.

## Data Availability

All relevant supplemental data that support the findings of study are available within the supporting table S1-S8.

## Appendix A Supplementary data

Supplementary material Image 1
