# Supplementary material for: Comprehensive quality evaluation for oysters of geographical indication from Rushan in China: Characteristic profile of nutrition and flavor components
Source: Food Chem X. 2025 Feb 25;26:102320. doi: 10.1016/j.fochx.2025.102320 (PMC11915176; doi:10.1016/j.fochx.2025.102320)
Supplement: Supplementary file 1 — Supplementary material [file mmc1.docx]

**Supplementary Materials**

**Table S1**

Biometric characteristics of oyster samples from three types in Rushan.

| Biometric characteristics | Diploid | Triploid | Fattening |
| --- | --- | --- | --- |
| Shell length (mm) | 107.30 ± 4.72 | 107.50 ± 4.22 | 107.70 ± 4.08 |
| Shell height (mm) | 59.40 ± 2.67 | 59.30 ± 2.95 | 59.70 ± 1.57 |
| Shell width (mm) | 35.20 ± 0.92 | 35.60 ± 2.01 | 36.70 ± 2.00 |
| Total weight (g) | 131.19 ± 10.81 | 133.73 ± 13.62 | 135.32 ± 9.06 |

Note：Mean ± standard deviation.

**Table S2**

Changes in Proximate composition of oyster samples from three strains in Rushan (dry weight, g/100 g).

| Index | Diploid | Triploid | Fattening |
| --- | --- | --- | --- |
| Moisture | 83.28 ± 2.81a | 83.11 ± 1.82a | 81.99 ± 1.89a |
| *Protein | 48.27 ± 2.50a | 45.02 ± 1.65b | 47.21 ± 3.20ab |
| *Lipid | 9.30 ± 0.67b | 10.02 ± 0.60a | 9.62 ± 0.68ab |

Values are presented as means ± SD. Values in a same column that do not share a same superscript are significantly different (*P* < 0.05). * indicates that it was measured with dry samples.

**Table S3**

The fatty acid composition of oyster samples from three strains in Rushan (dry weight, mg/100g).

| Fatty acids | Diploid | Triploid | Fattening |
| --- | --- | --- | --- |
| C4:0 | 51.85 ± 4.02a | 42.83 ± 3.12b | 39.18 ± 1.69c |
| C14:0 | 197.46 ± 9.86a | 200.77 ± 8.86a | 192.44 ± 9.38a |
| C15:0 | 26.25 ± 2.51a | 22.18 ± 1.71b | 23.39 ± 2.12b |
| C16:0 | 722.81 ± 40.64a | 621.43 ± 39.06c | 673.77 ± 46.09b |
| C17:0 | 35.71 ± 3.98ab | 36.27 ± 5.42a | 31.93 ± 1.77b |
| C18:0 | 127.69 ± 8.43a | 104.25 ± 7.77b | 109.65 ± 6.20b |
| SFA | 1161.78 ± 38.86a | 1027.72 ± 48.93b | 1070.36 ± 55.15b |
| C16:1 | 172.42 ± 9.70a | 121.28 ± 9.52c | 141.05 ± 12.56b |
| C18:1n9t | 35.56 ± 3.64a | 29.10 ± 4.34b | 31.73 ± 3.09b |
| C18:1n9c | 102.04 ± 4.11a | 85.87 ± 6.28b | 97.49 ± 3.73a |
| C20:1 | 75.55 ± 3.25a | 65.40 ± 8.68b | 54.86 ± 6.06c |
| MUFA | 385.58 ± 9.03a | 301.65 ± 14.50c | 325.12 ± 14.98b |
| C18:2n6c | 61.73 ± 5.17b | 74.98 ± 2.81a | 64.44 ± 7.68b |
| C18:3n3 | 197.2 ± 10.03a | 163.30 ± 8.62b | 158.12 ± 6.00b |
| C20:4n6 | 86.40 ± 6.11b | 97.83 ± 6.84a | 73.84 ± 6.98c |
| C20:5n3(EPA) | 439.43 ± 13.31a | 406.78 ± 6.37b | 395.51 ± 12.22c |
| C22:6n3(DHA) | 679.24 ± 11.42a | 658.57 ± 9.45b | 630.09 ± 19.5c |
| PUFA | 1464.01 ± 26.99a | 1401.46 ± 17.27b | 1322 ± 26.49c |
| TFA | 3032.52 ± 518.16a | 2876.89 ± 602.99a | 2765.66 ± 450.62a |
| EPA+DHA | 1118.67 ± 17.86a | 1065.35 ± 9.01b | 1025.61 ± 21.28c |
| EFA | 258.93 ± 12.52a | 238.28 ± 9.10b | 222.56 ± 8.41c |
| n-3 PUFA | 1315.88 ± 22.76a | 1228.65 ± 11.64b | 1183.73 ± 22.04c |
| n-6 PUFA | 148.13 ± 9.25b | 172.81 ± 7.96a | 138.28 ± 12.33c |
| n-3/n-6 | 8.91 ± 0.52a | 7.12 ± 0.29b | 8.63 ± 0.80a |
| AI | 0.82 ± 0.03b | 0.84 ± 0.04b | 0.88 ± 0.04a |
| TI | 0.25 ± 0.01a | 0.24 ± 0.01b | 0.26 ± 0.01a |

Values are presented as means ± SD.

Values in a same column that do not share a same superscript are significantly different *(P* < 0.05).

**Table S4**

Hydrolyzed amino acid contents of oyster samples from three strains in Rushan (dry weight, g/100 g).

| Amino acids | Diploid | Triploid | Fattening |
| --- | --- | --- | --- |
| *Leucine | 2.66 ± 0.18a | 2.58 ± 0.18a | 2.75 ± 0.19a |
| *Isoleucine | 1.70 ± 0.10b | 1.65 ± 0.08b | 1.89 ± 0.07a |
| *Lysine | 1.32 ± 0.14a | 1.29 ± 0.11a | 1.32 ± 0.11a |
| *Methionine | 0.91 ± 0.06b | 0.91 ± 0.08b | 0.99 ± 0.10a |
| *Phenylalanine | 1.37 ± 0.09a | 1.33 ± 0.08a | 1.42 ± 0.14a |
| *Threonine | 2.07 ± 0.11b | 1.99 ± 0.13b | 2.24 ± 0.12a |
| *Valine | 1.61 ± 0.09a | 1.60 ± 0.07a | 1.70 ± 0.16a |
| EAA | 11.64 ± 0.66b | 11.35 ± 0.57b | 12.31 ± 0.69a |
| Arginine | 3.10 ± 0.14a | 2.88 ± 0.17b | 3.17 ± 0.14a |
| Glycine | 2.28 ± 0.15a | 2.24 ± 0.11a | 2.26 ± 0.18a |
| Proline | 1.23 ± 0.02b | 1.24 ± 0.03ab | 1.27 ± 0.04a |
| Tyrosine | 1.23 ± 0.06a | 1.21 ± 0.07a | 1.27 ± 0.12a |
| Alanine | 2.30 ± 0.16b | 2.25 ± 0.15b | 2.58 ± 0.14a |
| Aspartic acid | 3.49 ± 0.16a | 2.54 ± 0.15c | 2.91 ± 0.20b |
| Glutamic acid | 5.97 ± 0.14b | 5.81 ± 0.16c | 6.17 ± 0.14a |
| Histidine | 0.72 ± 0.04b | 0.72 ± 0.04b | 0.77 ± 0.05a |
| Serine | 1.98 ± 0.12ab | 1.93 ± 0.12b | 2.06 ± 0.11a |
| Cysteine | 0.06 ± 0.01a | 0.06 ± 0.02a | 0.05 ± 0.02a |
| NEAA | 22.36 ± 0.67a | 20.88 ± 0.64b | 22.51 ± 0.51a |
| TAA | 34.00 ± 1.29a | 32.23 ± 1.16b | 34.82 ± 1.13a |
| EAA/TAA (%) | 34.22 ± 0.73b | 35.19 ± 0.70a | 35.34 ± 0.96a |

Values are presented as means ± SD.

Values in a same column that do not share a same superscript are significantly different (*P* < 0.05).

* indicates that it was essential amino acid.

**Table S5**

Vitamin contents of oyster samples from three strains in Rushan (dry weight).

| Vitamins | Diploid | Triploid | Fattening |
| --- | --- | --- | --- |
| Vitamin A (µg/100 g) | 200.96 ± 11.63a | 212.34 ± 12.65a | 171.51 ± 14.50b |
| Vitamin E (mg/100 g) | 1.30 ± 0.13c | 2.36 ± 0.10a | 2.08 ± 0.10b |
| Vitamin D_2_ (µg/100 g) | 12.99 ± 0.69a | 6.13 ± 0.52c | 8.11 ± 0.60b |
| Vitamin D_3_ (µg/100 g) | 4.90 ± 0.34b | 5.68 ± 0.40a | 5.04 ± 0.35b |
| Vitamin B_1_ (µg/100 g) | 7.85 ± 0.30a | 6.50 ± 0.39b | 6.59 ± 0.74b |
| Vitamin B_2_ (µg/100 g) | 148.32 ± 8.42a | 102.19 ± 3.89c | 130.47 ± 4.25b |
| Vitamin B_3_ (mg/100 g) | 3.68 ± 0.23a | 3.11 ± 0.16c | 3.47 ± 0.22b |
| Vitamin B_5_ (µg/100 g) | 318.46 ± 23.55a | 240.44 ± 31.63c | 292.28 ± 23.23b |
| Vitamin B_6_ (µg/100 g) | 14.49 ± 1.10a | 14.18 ± 0.81a | 14.13 ± 0.22a |
| Vitamin B_7_ (µg/100 g) | 37.25 ± 1.98a | 21.31 ± 0.91b | 15.00 ± 0.84c |

Values are presented as means ± SD.

Values in a same column that do not share a same superscript are significantly different (*P* < 0.05)

**Table S6**

Mineral concentrations of of oyster samples from three strains in Rushan (dry weight).

| Elements | Diploid | Triploid | Fattening |
| --- | --- | --- | --- |
| Ca (mg/g) | 6.84 ± 0.26b | 8.12 ± 0.67b | 8.21 ± 0.34a |
| P (mg/g) | 5.71 ± 0.42a | 5.18 ± 0.37b | 5.66 ± 0.52a |
| K (mg/g) | 12.28 ± 0.59a | 12.04 ± 0.37a | 12.04 ± 0.73a |
| Na (mg/g) | 23.11 ± 2.08a | 20.78 ± 2.79b | 19.12 ± 2.07b |
| Mg (mg/g) | 3.10 ± 0.33a | 2.79 ± 0.24b | 2.62 ± 0.22b |
| Fe (mg/kg) | 765.57 ± 26.26a | 719.27 ± 36.35b | 484.08 ± 22.85c |
| Zn (mg/kg) | 1299.14 ± 23.52a | 711.06 ± 35.96c | 851.49 ± 24.66b |
| Se (mg/kg) | 6.23 ± 0.74a | 5.90 ± 0.32a | 6.13 ± 0.24a |
| Cu (mg/kg) | 513.73 ± 33.92a | 336.59 ± 23.54b | 361.09 ± 28.4b |
| Mn (mg/kg) | 41.38 ± 2.67a | 34.67 ± 1.63b | 29.45 ± 2.08c |

Values are presented as means ± SD.

Values in a same column that do not share a same superscript are significantly different (*P* < 0.05).

**Table S7**

Nutritional quality indexes of oyster samples (dry weight).

| Indices | RNI | INQ | | |
| --- | --- | --- | --- | --- |
|  |  | Diploid | Triploid | Fattening |
| Vitamin A (µg/100 g) | 770 | 2.29 | 2.39 | 1.99 |
| Vitamin D (mg/100 g) | 10 | 15.53 | 10.26 | 11.40 |
| Vitamin E (mg/100 g) | 14^#^ | 0.85 | 1.47 | 1.27 |
| Vitamin B_1_ (mg/100 g) | 1.4 | 0.05 | 0.04 | 0.04 |
| Vitamin B_2_ (mg/100 g) | 1.4 | 0.92 | 0.63 | 0.81 |
| Vitamin B_3_ (mg/100 g) | 15 | 2.13 | 1.80 | 2.01 |
| Vitamin B_5_ (µg/100 g) | 5^#^ | 0.55 | 0.42 | 0.51 |
| Vitamin B_6_ (mg/100 g) | 1.4 | 0.09 | 0.09 | 0.09 |
| Vitamin B_7_ (mg/100 g) | 40^#^ | 8.08 | 4.62 | 3.25 |
| Ca (mg/100 g) | 800 | 7.42 | 8.81 | 8.91 |
| P (mg/100 g) | 720 | 6.88 | 6.24 | 6.82 |
| K (mg/100 g) | 2000^#^ | 5.33 | 5.23 | 5.22 |
| Na (mg/100 g) | 1500^#^ | 13.37 | 12.02 | 11.06 |
| Mg (mg/100 g) | 330 | 8.16 | 7.33 | 6.89 |
| Fe (mg/100 g) | 12 | 55.36 | 52.02 | 35.01 |
| Zn (mg/100 g) | 12.5 | 90.19 | 49.37 | 59.12 |
| Se (µg/100 g) | 60 | 90.12 | 85.28 | 88.60 |
| Cu (mg/100 g) | 0.8 | 44.89 | 37.61 | 31.95 |
| Mn (mg/100 g) | 4.5^#^ | 99.07 | 64.91 | 69.64 |

Note: Reference intakes are calculated based on the recommended intakes or appropriate intakes for 18-50 years old males, # adequate intake.

**Table S8**

Nutritional quality indexes of oyster samples from three strains in Rushan (dry weight).

| Indices | INQ | | |
| --- | --- | --- | --- |
|  | Diploid | Triploid | Fattening |
| Protein (g/100 g) | 6.44 | 6.01 | 6.30 |
| SFA (g/100 g) | 17.12 | 16.25 | 17.26 |
| n-6 PUFA (mg/100 g) | 0.00 | 0.00 | 0.00 |
| n-3 PUFA (mg/100 g) | 0.22 | 0.22 | 0.22 |
| EPA+DHA (mg/100 g) | 1.32 | 1.35 | 1.33 |
